# Nutmeg oil alleviates chronic inflammatory pain through inhibition of COX-2 expression and substance P release *in vivo*

**DOI:** 10.3402/fnr.v60.30849

**Published:** 2016-04-26

**Authors:** Wei Kevin Zhang, Shan-Shan Tao, Ting-Ting Li, Yu-Sang Li, Xiao-Jun Li, He-Bin Tang, Ren-Huai Cong, Fang-Li Ma, Chu-Jun Wan

**Affiliations:** 1 Department of Pharmacology, College of Pharmacy, South-Central University for Nationalities, Wuhan, PR China; 2 Functional Oil Laboratory Associated by Oil Crops Research Institute, Chinese Academy of Agricultural Sciences and Infinitus (China) Company Ltd., Guangzhou, PR China; 3 Oil Crops Research Institute, Chinese Academy of Agricultural Sciences, Wuhan, PR China

**Keywords:** nutmeg oil, chronic pain, allodynia, COX-2, substance P

## Abstract

**Background:**

Chronic pain, or sometimes referred to as persistent pain, reduces the life quality of patients who are suffering from chronic diseases such as inflammatory diseases, cancer and diabetes. Hence, herbal medicines draw many attentions and have been shown effective in the treatment or relief of pain.

**Methods and Results:**

Here in this study, we used the CFA-injected rats as a sustainable pain model to test the anti-inflammatory and analgesic effect of nutmeg oil, a spice flavor additive to beverages and baked goods produced from the seed of *Myristica fragrans* tree.

**Conclusions:**

We have demonstrated that nutmeg oil could potentially alleviate the CFA-injection induced joint swelling, mechanical allodynia and heat hyperanalgesia of rats through inhibition of COX-2 expression and blood substance P level, which made it possible for nutmeg oil to be a potential chronic pain reliever.

It is always unfortunate to be suffering from different chronic diseases such as inflammatory diseases, cancer, and diabetes. However, persistent pain (or chronic pain) makes conditions of those patients even worse and significantly reduces their quality of life. Hence, in clinical investigation, a great challenge is to discover safer, more effective and better-tolerated analgesics (1, 2). However, side effects such as nausea, sedation, itching, liking, and disliking of the drug and respiratory depression usually come with commercially available analgesics (3, 4). Thus, extensive research and development efforts have been directed toward the discovery of novel analgesics in both academia and industry.

Recently, many researchers have demonstrated that the herbal medicines (phytomedicine) are effective in the treatment or relief of pain, such as *Aleurites moluccana* (L.) Willd leaves extract (5), flower extract of *Tanacetum parthenium* (6), and extract of 25 more plant species traditionally used for pain relieving (7).

Nutmeg is commonly served in powder form or essential oil in the food industry worldwide. It has been reported that nutmeg oil, commercially obtained by steam distillation from kernels of nutmeg, usually contains more than 50 chemical components such as linalool, terpineol, eugenol, myristicin, camphene, dipentene, and pinene (8). Although some concerns about the toxicity of its components have been published (9, 10), lots of research still focused on its excellent activities in radical removal, lipid oxidation inhibition (11, 12), and most importantly its anti-inflammatory and antimicrobial activities (8, 13–15). Externally, nutmeg oil can also be used for rheumatic pain and, like clove oil, can be applied as an emergency treatment to dull toothache.

So far, no study has been carried out to evaluate the combined effects of nutmeg oil on inflammation and pain. Therefore, an inflammatory pain model of rat has been used in this study and three different evaluation methods have been introduced to test the anti-inflammatory and analgesic effects of nutmeg oil. Moreover, the expression levels of cyclooxygenase-2 (COX-2), a key molecule induced as an early response to pro-inflammatory mediators and stimuli such as endotoxins and cytokines (16), and blood level of substance P, a key neurotransmitter and neuromodulator in pain perception (17, 18), have also been investigated in skin samples of the model rats.

## Materials and methods

### Reagents

The complete Freund's adjuvant (CFA) was purchased from Sigma (F5881, Sigma-Aldrich, USA). Diclofenac was purchased from Novartis, Beijing. The nutmeg oil and Gas chromatography–mass spectrometry (GC–MS) results of nutmeg oil were kindly provided by the Functional Oil Laboratory Associated by Oil Crops Research Institute, Chinese Academy of Agricultural Sciences and Infinite Co., LTD., Guangzhou 510000, China. 2% tween-20 was used for emulsification of nutmeg oils and diluted before the application. All other reagents were purchased from Sigma-Aldrich, USA.

### Distilling of nutmeg oil

Nutmeg oil used in the study was distilled from dried seeds of *Myristica fragrans Houtt* (produced in Guangdong, China). Briefly, dried seeds (purchased from Chinese medicine Yinpian factory, Guangzhou medicine company, Guangzhou, China) were pulverized and mixed with water in a ratio of 1 g to 10 mL. Subsequently, the nutmeg oil was obtained by distilling the mixture in steam distillation apparatus for 8 h until no oil drop was seen in distilled fraction.

### GC–MS analysis of nutmeg oil

Nutmeg oil (0.103 g) was placed in a 10-mL volumetric flask with n-Hexane (chromatographically pure) as a solvent. The solution of nutmeg volatile oil (0.5 µL) was inserted into the GC injector. GC–MS analyses were performed using an Agilent 7890 (II) GC coupled to an Agilent 5975 series mass selective detector operating in the electron impact ionization mode at 70 eV with a mass range of 50–450 m/z. Volatile compounds were separated using an HP-5MS capillary column (polydimethylsiloxane 5% diphenyl, 30 m×0.32 mm×0.25 µm, Hewlett Packard). The temperature was programmed from 40°C (1 min) to 220°C at 5°C/min, with a final 1 min hold. Helium was used as the carrier gas, with a volumetric flow rate of 1.0 mL/min. For the analyses of volatile oil of nutmeg, split mode was used with a ratio of 1:60 and an injector temperature of 240°C. All samples were analyzed in triplicate. Retention indices (RI) were calculated using an n-alkane series. The components were identified by the comparison of their RI relative to C4-C30 n-alkanes (Sigma Chemical Co., St. Louis, MO) that were obtained from the HP-5MS column and compared to the data provided by the NIST mass spectral libraries. Positive identification was assumed when a good match of the mass spectrum and RI was achieved.

### Animals

Young male Wistar rats (190–250 g) were acclimatized for 7 days under specific pathogen-free conditions before the initiation of experiments. The animals were kept in a temperature-controlled laboratory (22–25°C) with a 12-h light–dark cycle. The care and use of animals for this study was performed according to the Guide for Animal Experimentation, South-Central University for Nationalities and the Committee of Research Facilities for Laboratory Animal Sciences, South-Central University for Nationalities, China. The protocols were approved by the Committee on the Ethics of Animal Experiments of the South-Central University for Nationalities, China (Permit Number: 2013-SCUEC-AEC-002). All efforts were made to minimize suffering.

### Inflammatory pain model

**Fig. 1:**
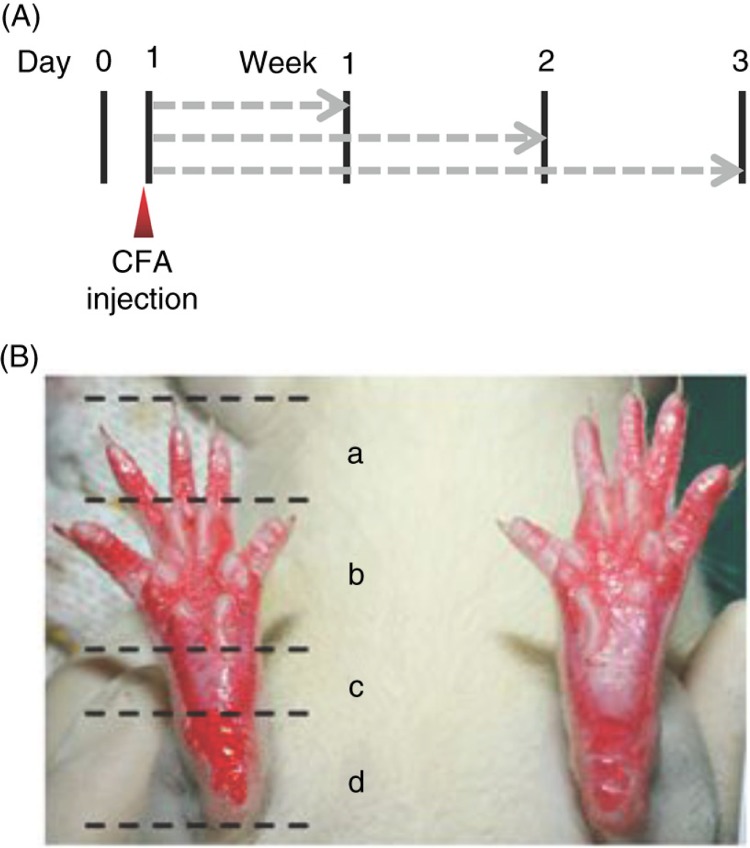
(A) Schedule of the animal experiment. Vertical bars, assessments and tests at different time point (indicated above); dashed arrows (in gray), different treatments (frequency: daily). (B) Representative graph of both hind paws of a rat during the pain score test. (a) Toes; (b) anterior sole; (c) posterior sole; (d) heel. The color of all four areas of the right paw was deeper than the left paw and hence scored 4 in this case.

Rats were randomly divided into control group (no CFA treatment) and CFA-induced inflammatory pain group (CFA treatment). The detailed sample sizes of different groups are included in the Results section. Inflammatory pain was induced by a subcutaneous injection of 150 µl CFA into the bottom of the left hind paws of rats. The control rats were injected with the same volume of saline. After the CFA injection, rats in control group were randomly divided into three groups (control) and sacrificed in the end of the first, second, or third week, respectively. The CFA group were randomly divided into CFA-treated only (CFA), CFA-treated with continuous oral administration of diclofenac sodium (CFA+diclo, 30 mg/kg/day, Novartis, Beijing) afterward, and CFA-treated with continuous oral administration of high-dose nutmeg oil (CFA+NOhigh, 20 mg/kg/day) and low-dose nutmeg oil (CFA+NOlow, 10 mg/kg/day). These rats were sacrificed in the end of the first, second, or third week as well. The detailed schedule was shown in Fig. 1A.

### Assessment of inflammation

The extent of inflammation was assessed by paw swelling. The paw volume was monitored with a water displacement plethysmometer (PV-200; Chengdu Taimeng Software Co. Ltd., Chengdu, China), in triplicate. Then, the paw swelling was expressed as the ratio of the volume of the right paw for each rat to the volume of its left paw. Data acquired in the end of the first, second, and third week after the CFA injection were presented in percentage for the control group and treated groups.

### Pain score test

All behavioral assessments were performed under the ethical guidelines by the International Association for the Study of Pain (IASP). All of the behavioral tests were conducted blindly. All behavioral tests were performed at the following time points: 1, 2, or 3 weeks after the CFA injection. To eliminate diurnal rhythm, these tests were performed at the same time of the day. The pain score test was performed as follows: both the hind paws of the rat were uniformly marked with red ink. In a self-made semi-enclosed rat-walking trail, marked rats were allowed to walk for a distance of 0.5 m, and photos of both hind paws were taken. Subsequently, five persons were asked to grade the pain scores independently and double-blinded with the following criteria: compared with the left paw, a deeper color in any of the four areas (a, toes; b, anterior sole; c, posterior sole; d, heel) of the right paw scores 1, and hence, the rat in Fig. 1B got a score of 4.

### Mechanical allodynia test

Animals were placed on a metal mesh grid under a plastic chamber, and the tactile threshold was measured by applying a von Frey filament (North Coast Medical, Gilroy, CA, USA) to the mid-plantar surface of the hind paw until a positive response for withdrawal behavior was elicited. Different calibrated fine von Frey filaments (0.40, 0.70, 1.20, 2.00, 3.63, 5.50, 8.50, 15.1, and 21.0 g) were presented serially to the hind paw in ascending order of strength with sufficient force to evoke slight bending. A brisk paw withdrawal response was considered as a positive response. If there was no response, the next filament with greater force would be tested. When no response was observed at 21 g of pressure, the animal was recognized as being at the cut-off value. The 50% withdrawal response threshold was determined using the up–down method (19).

### Thermal hyperalgesia test

To determine nociceptive responses to heat stimuli, a previously described paw withdrawal response latency (WRL) measurement was used (20). In brief, animals were placed in a plastic chamber with a glass floor and allowed to acclimatize for 10 min. A radiant heat source was placed under the glass floor beneath each hind paw, and paw WRL was measured to the nearest 0.1 sec using a plantar tester (Ugo Basile Srl, Varese, Italy). The intensity of the light source was calibrated to produce a paw withdrawal response within a range of 16–25 sec in naive animals. The test was examined in triplicate on both the ipsilateral and contralateral hind paws, and the mean WRL in each hind paw was calculated. The cut-off time was set at 60 sec.

### Blood substance P detection

Rats with different treatments were sacrificed 1, 2, or 3 weeks after the CFA-injection, respectively. The blood samples were collected and pipetted into a disposable test tube with EDTA and PI (peptidase inhibitors; 1 µM phosphoramidon, 4 µg/mL bacitracin and 1 µM captopril; Sigma Chemical Co., St Louis, MO, USA). After 1 h, the blood plasma was collected and the substance P content in the blood was measured using a highly sensitive radio-immuno-assay as previously described (21).

### Skin biopsy

The skin specimens with diameter of 3 mm around the CFA-injected regions were collected and fixed immediately in 10% phosphate-buffered formalin solution for 16–24 h, embedded in paraffin and cut into 4-µm-thick sections and followed by hematoxylin and eosin (H&E) or Masson's trichrome staining by standard techniques. Histopathologic examinations of the liver sections were conducted using a Nikon 50i light microscope (Nikon Inc, Tokyo, Japan).

### Immunohistochemical staining and multispectral imaging analysis of COX-2 expression

Briefly, skin sections from different treatments were incubated with mouse anti-COX-2 primary antibody (dilution of 1:300, CX229, Cayman chemical, Michigan, USA) overnight at 4°C. After washing, the sections were incubated with appropriate biotin-conjugated secondary antibody (dilution of 1:100; Santa Cruz Biotechnology, CA, USA) for 30 min at room temperature. The color development (brown) was performed using a DAB substrate kit (Nichirei, Japan), and the sections were counterstained with hematoxylin (blue). Then, multispectral imaging analysis of sections was performed by using a Nikon 50i light microscope (Nikon Inc, Tokyo, Japan) with a Nuance Multispectral Imaging System (Cambridge Research and Instrumentation Inc., Woburn, MA) according to the method instructions (22).

### Statistical analysis

All results of behavior tests (except otherwise instructed) in the paper were presented as percentages between the CFA-injected paws and control paws in the form of mean±SD. Statistical analysis was carried out by a one-way analysis of variance followed by Bonferroni post-tests in Origin8.0 (Originlab Corp., MA, USA). All tests were two-tailed, *P*<0.05 were considered to be statistically significant, and in all graphs, *, **, or *** represents *P*<0.05, 0.01, or 0.001, respectively. The results of immunostaining and substance P detection were presented as mean±SEM.

## Results

### Identifying the composition of nutmeg oil

**Fig. 2:**
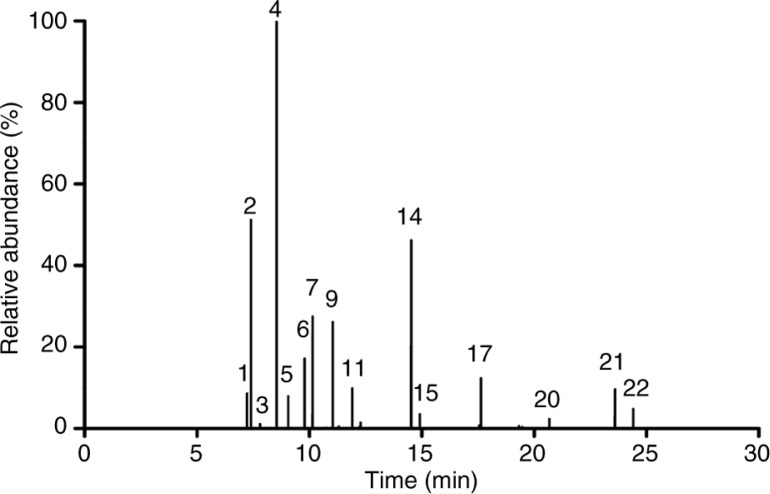
GC–MS analysis of the nutmeg oil. The detailed information of chemical composition was included in Table 1.

The major compounds of the nutmeg oil used in this study were sabinene (25.00%), 4-terpineol (11.54%), α-pinene (12.79%), limonene (6.87%), and γ-terpinene (6.52%). The detailed data describing all the compounds are presented in Fig. 2 and Table 1 in comparison of a recent study (23).

**Table 1:** Chemical composition of the nutmeg oil

No.	Retention time	LRI	% exp^a^	% ref^b^	Compounds
1	7.224	868	2.15	0.78	α-Thujene
2	7.399	910	12.79	10.23	α-Pinene
3	7.806	984	0.27	0.16	Camphene
4	8.544	1,137	25.00	21.38	Sabinene
5	9.06	1,269	1.98	2.38	α-Myrcena
6	9.782	1,423	4.28	2.72	α-Terpinene
7	10.141	1,502	6.87	5.57	Limonene
8	10.742	1,595	0.04	0.03	*trans*-β-Ocimene
9	11.039	1,683	6.52	3.98	γ-Terpinene
10	11.307	1,755	0.12	0.06	*trans*-Sabinene hydrate
11	11.907	1,872	2.45	1.62	Terpinolene
12	12.225	1,924	0.08	0.06	*cis*-Sabinene hydrate
13	12.28	1,967	0.35	0.75	Linalool
14	14.53	2,423	11.54	13.92	4-Terpineol
15	14.915	2,526	0.87	3.11	α-Terpineol
16	17.56	3,094	0.17	0.24	Bornyl acetate
17	17.632	3,114	3.07	4.28	Safrole
18	19.331	3,482	0.14	0.77	Citronellol
19	19.481	3,511	0.09	1.74	Isoeugenol
20	20.686	3,765	0.74	1.74	Methyl eugenol
21	23.598	4,383	2.40	13.57	Myristicin
22	24.403	4,563	1.20	1.42	Elimicin

a Compound percentage in the current study.

b Compound percentage in previous study (23).

### Application of nutmeg oil alleviated CFA-injection-induced joint swelling of rats

**Fig. 3:**
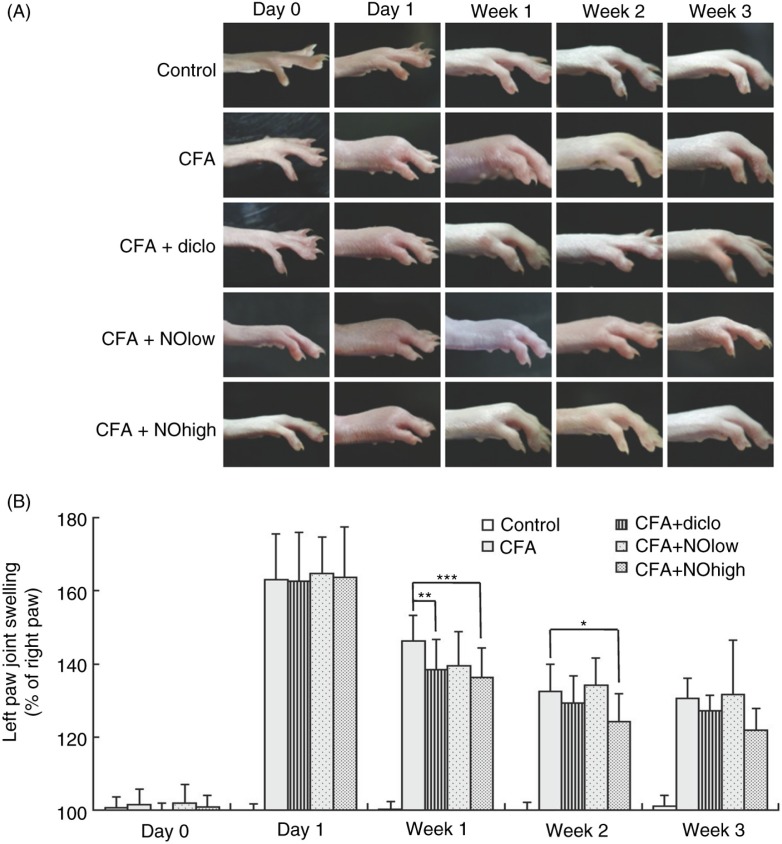
The levels of joint swelling in different groups of rats. (A) Representative graphs of left hind paws at different time points under different treatments. (B) Summary of the joint swelling of the left hind paws (presented as percentage of the right paw for each rat). CFA, the complete Freund's adjuvant; diclo, sodium diclofenac; NOlow, low concentration of nutmeg oil; NOhigh, high concentration of nutmeg oil. *, **, *** represent *P*<0.05, 0.01, 0.001, respectively. Data were presented as mean±SD.

To evaluate the anti-inflammatory and analgesic effects of nutmeg oil, CFA-injected rats model was used in our study as instructed in the Materials and Methods section. As shown in Fig. 3A, before the CFA-injection, no swelling was observed in left hind paws of each group. However, after the CFA-injection, the injected hind paws of rats displayed a certain level of swelling compared with the control group (Fig. 3B, in percentage: 163.05±12.69 of CFA-injected group, *n*=18 vs. 99.24±2.47 of control group, *n=*24), and the extent of swelling decreased in a time-dependent manner. With the application of different treatment (diclo, sodium diclofenac as positive control, 30 mg/kg/day, *n=*22; NOlow, low dose of nutmeg oil, 10 mg/Kg/day equivalent to 100 mg/day for a 60-Kg weight human, *n=*12; NOhigh, high dose of nutmeg oil, 20 mg/Kg/day, *n=*17), the extent of swelling decreased more rapidly compared with the CFA group. More importantly, in the presence of high dose nutmeg oil, the decrease was much more significant than diclofenac-treated group (week 1, 27.43% of NOhigh vs. 24.17% of diclo; week 2, 39.51% of NOhigh vs. 33.29% of diclo; week 3, 41.90% of NOhigh versus 35.52% of diclo).

### Application of nutmeg oil partially restored pain sensation of CFA-injected rats

**Fig. 4:**
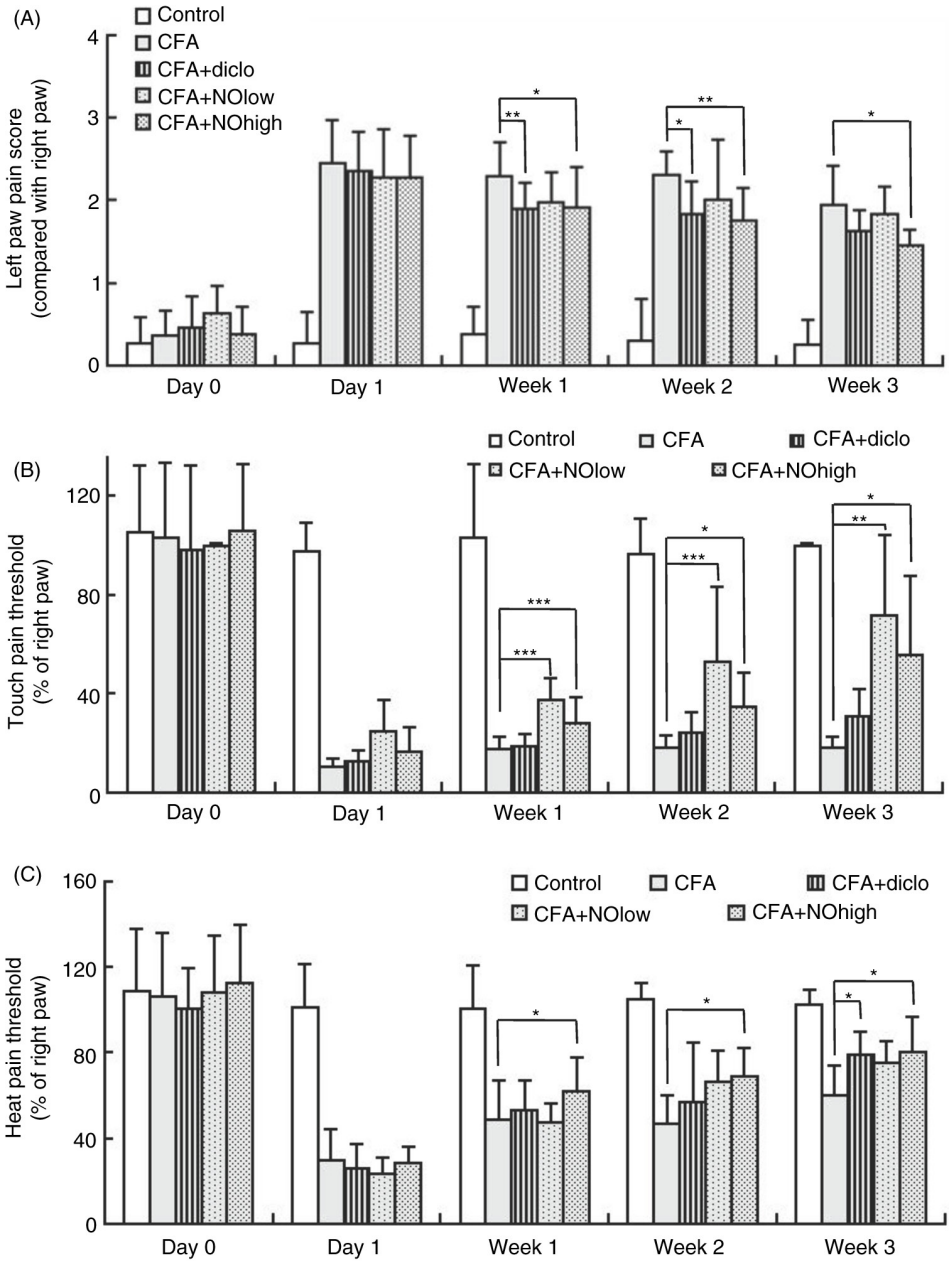
Pain behavioral tests of different groups of rats at different time points under different treatments. (A) Summary of the results of pain score test. (B) Summary of the results of touch pain threshold measurement. (C) Summary of the results of heat pain threshold measurement. Data were presented as mean±SD.

To better characterize the inflammation-associated pain states of rats under different treatment, pain score test was introduced in the current study. The detailed procedure of pain score test was in the Materials and Methods section. As summarized in Fig. 4A, the CFA injection increased the pain score for ~7-folds from 0.37±0.30 at day 0 to 2.45±0.52 at day 1 and remained in a relatively high level (1.95±0.46 at week 3) for as long as 3 weeks, suggesting that the CFA injection successfully induced a significant and sustainable pain to hind paws of rats. With the application of diclofenac, the positive control drug, pain score dropped to 1.89±0.32 at week 1 and further decreased to 1.625±0.25 at week 3. However, a high dose of nutmeg oil treatment reduced the pain score to 1.90±0.51 at week 1 and 1.46±0.19 at week 3, which surpass the effect of diclofenac application, suggesting that high dose application of nutmeg oil showed a better effect of alleviating the inflammatory pain response induced by the CFA injection.

The pain score test was conducted by five persons independently and double-blindly. However, there could still be some bias and subjectivity during the experiments. In addition, the score demonstrated in this test was an overall and complex readout of pain behavior. Hence, a traditional touch pain test and heat pain test was also used to test the anti-inflammatory and analgesic effects of nutmeg oil. As shown in Fig. 4B, we observed a significant sensitization of both touch and heat induced pain response after the CFA injection. In accordance with our previous results, both the low- and high-dose treatment of nutmeg oil alleviated the hypersensitivity of touch pain behavior of CFA-injected rats. The touch pain threshold enlarged about 3-folds from (in percentage) 24.72±12.63 at day 1 to 71.67±32.72 at week 3 for low dose application of nutmeg oil and from 16.19±10.29 at day 1 to 55.71±31.78 at week 3 for high dose. In the contrary, the threshold for CFA group merely increased from 10.09±3.73 at day 1 to 18.33±4.27 at week 3 suggesting a poor self-recovery on the allodynia. It is interesting to note that the positive control drug, sodium diclofenac, showed less potency in raising touch pain threshold compared with nutmeg oil (from 12.50±4.71 at day 1 to 30.83±10.73 at week 3). Consistent with the touch pain test result, heat pain thresholds were also greatly reduced by the CFA injection and restored by high-dose treatment of nutmeg oil (from 28.49±7.37 at day 1 to 80.22±16.67 at week 3). However, in our experiments, the effects of low-dose treatment of nutmeg oil or diclofenac on alleviating the heat hyperanalgesia were merely observable (Fig. 4C).

### Application of nutmeg oil inhibited COX-2 expression and substance P release in CFA-injected rats

**Fig. 5:**
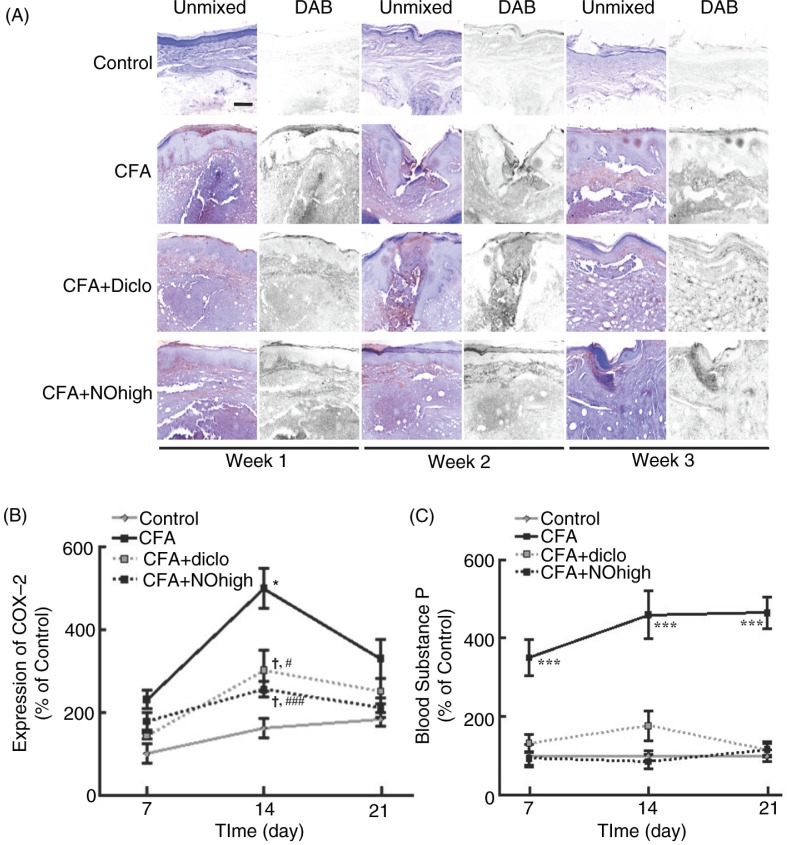
Nutmeg oil alleviates pain via inhibition of COX-2 expression and reduction of blood Substance P level. (A) Representative graphs of COX-2 expression in CFA-injected area of rats at different time points under different treatments. Scale bar: 100 µm. (B) Summary of COX-2 expression. *, significantly different from control group; †, not different from control group; #, ###, significantly different from CFA group with *P* <0.05, 0.001, respectively. (C) Summary of substance P level in blood at different time points under different treatments. ***, significantly different from control group with *P*<0.001. Data were presented as mean±SEM.

It has been demonstrated that CFA-injection could induce COX-2 expression in rats (24–26). To test whether pain alleviation of nutmeg oil application was mediated by inhibition of COX-2, skin samples around the CFA-injected regions were collected and conducted to immunohistochemistry staining. As shown in Fig. 5A and B, the expression pattern of COX-2 varied after diclofenac or high dose of nutmeg oil treatment. Both treatments decreased COX-2 expression induced by the CFA injection, especially at week 2. Moreover, substance P release has been demonstrated to be correlated with pain response in rats (22). Hence, the blood substance P levels were investigated in the current study as summarized in Fig. 5C. Compared with CFA-treated rats, diclofenac application could greatly diminish CFA-injection induced increase of blood substance P level, while application of high-dose nutmeg oil nearly abolished the rise, suggesting a better analgesic effect compared with diclofenac.

## Discussion

Nutmeg is commonly used as spice flavor additives to beverages and baked goods produced from the seed of *Myristica fragrans* tree. However, large amounts intake could result in toxic effects such as nausea, vomiting, or hallucination. Reports have correlated toxicities of nutmeg with myristicin, safrole, and elemincin (27). Therefore, the content of myristicin and safrole in nutmeg oil is required to be around 5–10% (23). In our study, the total content of safrole (3.07%), myristicin (2.40%), and elemincin (1.20%) is less than 10%. During the experiments, we didn't observe any abnormality of rats after nutmeg oil application, and hence, the nutmeg oil used in the study should be safe for at least animal experiments. For further human usage, more experiments of toxicity and safety should be conducted.

Monoterpenes have been demonstrated to be major compounds in many essential oils extracted from different plants and could be useful in many conditions and symptoms including premenstrual problems, mastalgia, inflammation, sexual dysfunction, and pain (28–30). Monoterpenes can be subdivided into three categories: acyclic monoterpenes (citronellal, citronellol, and linalool), monocyclic monoterpenes (limonene), and bicyclic monoterpenes (pinene, carene, sabinene, camphene, and thujene), which are abundant in the nutmeg oil used in the current study (sabinene: 25%, α-pinene: 12.79%, 4-terpineol: 11.54%, limonene: 6.87%, γ-terpinene: 6.52%, α-terpinene: 4.28%). Hence, it would not be too surprising that nutmeg oil could alleviate inflammatory pain induced by the CFA injection after oral application.

The CFA-injection in the present study successfully produced avoidance responses during heat and touch stimulation (the pain score test detected the retrieval behavior of rats during walking which can be treated as touch sensation as well), indicating that the CFA-injection resulted in affective pain as previously described (26, 31). However, unlike the previous studies, we detected a relatively long disturbance of allodynia and heat hyperanalgesia, for 3 weeks after CFA-injection in this study. Our data showed that the influence of CFA-injection can last at least for 3 weeks since most of our test results at week 3 didn't recover to the control level. Moreover, it is also quite interesting to note that the CFA-injection-induced allodynia was much worse than thermal (particularly heat) hyperanalgesia, since touch pain thresholds remains to be quite low after recovered for 3 weeks from the CFA-injection while heat pain thresholds restored to ~70%.

Diclofenac, which belongs to the nonsteroidal anti-inflammatory drugs, is probably the most commonly used pain reliever (32). As an anti-inflammatory and analgesic drug, it is probably the most efficient inhibitor of prostaglandins, which are used for treatment of rheumatic diseases (33) and post-operational pain relievers (34). However, side effects such as headache, dizziness, rash, edema, and potential damage on kidney and liver restricted its clinical usage, and hence, many studies have tried other substitutions such as B and E vitamins (35, 36). Apart from the side effects, diclofenac (30 mg/kg/day) is not that potent in reducing chronic pains as observed in our study. In contrast, both low- and high-dose nutmeg oil worked better in relieving allodynia. It is also intriguing that low-dose nutmeg oil seems to be more powerful in recovering allodynia caused by the CFA injection. We speculated that this could be due to different underlining mechanism of nutmeg oil toward mechanical or heat hyperanalgesia, which needs to be further investigated.

For many years, COX-2 has been thought to play a central role in inflammation-induced pain, and studies have already shown that the COX-2 protein and mRNA expression level is elevated acutely as soon as 6 h after CFA-injection (26). However, in this study we have demonstrated that this elevation of COX-2 can last at least for 2 weeks (Fig. 5B) at the injection region, and this prolonged elevation is sensitive to both diclofenac and nutmeg oil treatment, suggesting a potential role for nutmeg oil in relieving chronic inflammation and pain.

The neuropeptide substance P belongs to the tachykinin family, which has been shown to be functional in regulating biological functions such as emotional stress, neurogenic inflammation, alcohol addiction, mitogenesis, angiogenesis, emesis, pain, chemotaxis of leukocytes, and pruritus in a dose-dependent manner (37). Substance P has been shown to have the highest affinity for and be its natural ligand of NK-1 receptor. Evidences have indicated that NK-1 is important for inflammatory pain hypersensitivity (38, 39). The interaction of substance P and NK-1 and subsequent internalization of the complex are crucial for mechanical allodynia in inflammatory pain model (40). Here, we have shown that the blood substance P level increased for ~4-folds after the CFA injection and this increase was sensitive to nutmeg oil treatment, suggesting compounds in the nutmeg oil could potentially play a role in the aforementioned process, which need to be further screened and verified.

## Conclusion

We have demonstrated that nutmeg oil could potentially alleviate the CFA-injection-induced joint swelling, allodynia, and heat hyperanalgesia of rats through inhibition of COX-2 expression and blood substance P level, which made it possible for nutmeg oil to be a potential chronic inflammation and pain reliever.
